# Effect of Rennet Type on Selenium Retention and Its In-Vitro Bioaccessibility in Se-Enriched Edam Cheese: An Exploratory Study

**DOI:** 10.3390/foods15173123

**Published:** 2026-09-02

**Authors:** Lukáš Praus, Veronika Legarová, Lucie Kejdová Rysová, Michal Šrámek, Lukáš Kaplan, Ondřej Sedlář, Pavel Tlustoš, Jiřina Száková

**Affiliations:** 1 Department of Agroenvironmental Chemistry and Plant Nutrition, Czech University of Life Sciences, Kamýcká 129, Praha-Suchdol, 165 00 Prague, Czech Republic; sramekm@af.czu.cz (M.Š.); kaplanl@af.czu.cz (L.K.); sedlar@af.czu.cz (O.S.); tlustos@af.czu.cz (P.T.); szakova@af.czu.cz (J.S.); 2 Department of Food Science, Czech University of Life Sciences, Kamýcká 129, Praha-Suchdol, 165 00 Prague, Czech Republic; legarova@af.czu.cz (V.L.); rysoval@af.czu.cz (L.K.R.)

**Keywords:** cheesemaking technology, enzymatic coagulation, selenium speciation, selenomethionine quantification, gastrointestinal digestion, micronutrient bioavailability, functional food

## Abstract

Dairy products, particularly cheese from selenium-enriched milk, provide an effective vehicle for Se supplementation in the human diet. This study examined how coagulant type and ripening affect total Se (Se_tot_), free selenomethionine (SeMet) formation, and in vitro Se bioaccessibility in Edam-type cheeses produced from biofortified milk (73 µg L^−1^ Se). Cheeses were manufactured in a single batch using either animal (calf) or microbial (*Rhizomucor miehei*) rennet and ripened for six weeks under controlled laboratory conditions. Although Se_tot_ declined in both cheeses, microbial-rennet cheese exhibited a rapid 13.5% loss in week 1, whereas animal-rennet cheese showed a more gradual decrease. After six weeks, both cheeses retained about 40 µg Se per 100 g. A robust 20 mmol L^−1^ ammonium acetate extraction coupled with LC-ICP-MS enabled detection of free SeMet at concentrations as low as 0.0003 µg g^−1^ Se and revealed a linear increase throughout ripening in both cheeses. In vitro Se bioaccessibility reached 64% of Se_tot_ for animal-rennet cheese and approached complete release in microbial-rennet cheese. These findings suggest that the choice of coagulant may influence not only Se retention, but also the gastrointestinal accessibility of protein-bound Se in Edam-type cheese.

## 1. Introduction

Cheese produced from bovine milk is a widely consumed dairy product that contributes substantially to the dietary intake of essential nutrients, including selenium [1,2]. Selenium (Se) deficiency and suboptimal intake remain widespread nutritional concerns worldwide [3,4]. The adequate intake for adults has been set at 70 µg day^−1^ by the European Food Safety Authority (EFSA) [5], a level that dietary intakes in many regions do not consistently meet. The contribution of dairy products to meeting daily Se requirements varies across countries, influenced by dietary habits, individual preferences, product availability, and Se concentrations in raw milk [2,6,7]. In practice, commercial dairy farms maintain the Se status of herds primarily through direct supplementation of lactating cows with selenite (Se^IV^), selenate (Se^VI^), or seleno-yeast [8]. Milk Se concentration can vary substantially, with values ranging from 20 to 400 µg kg^−1^ and an average of 89 ± 84 µg kg^−1^ even within a single region of one country [9], mainly reflecting differences in supplementation practice.

Several studies have demonstrated that cheese can serve as an effective delivery system for Se in the human diet [1,10,11,12,13]. Human intervention trials with Se-enriched milk and dairy protein products have demonstrated significant increases in plasma Se and modulation of selenoprotein expression in consumers [14,15]. Research has also examined the effects of Se enrichment on cheese composition, nutraceutical properties, and quality attributes such as texture, functionality, and sensory characteristics [1,11,13], although these studies have almost exclusively quantified total Se rather than its speciation or bioaccessibility. However, the concept of producing Se-enriched cheese still lacks a mechanistic understanding of how cheesemaking technology shapes Se transformation during ripening, and in particular how variables such as rennet type influence not only Se retention in cheese, but also the nutritional quality and potential bioavailability of Se during maturation. In Se-enriched cheese, proteolysis is of particular interest because Se is an integral part of milk casein [12]. During ripening, cheese proteolysis is catalyzed by enzymes from the coagulant, indigenous milk enzymes, and enzymes from starter, nonstarter, and secondary cultures [16], providing multiple opportunities to influence proteolysis in cheesemaking. The effect of the coagulation agent on early hydrolysis of casein fractions (α-, β-, and κ-caseins) and subsequent peptide breakdown has been extensively studied [17,18,19]. Although numerous studies have characterized proteolysis products using conventional separation techniques such as liquid chromatography [20] and capillary electrophoresis [18,21], as well as advanced liquid chromatography with tandem mass spectrometry (LC-MS/MS) [22], direct determination of Se-containing peptides and amino acids in cheese is still rare [10,11].

Since microbial coagulants were previously shown to promote more extensive hydrolysis of αs- and β-caseins and higher free amino acid (FAA) formation than calf rennet [18], the present study uniquely investigated whether such differences in proteolytic pathways also affect Se retention, free selenomethionine (SeMet) formation dynamics, and Se bioaccessibility in ripened cheese. To address this question, a laboratory-scale ripening trial was performed using Se-enriched milk to manufacture semi-hard Edam-type cheese, a variety widely consumed in Central Europe. Specifically, the study aimed to: (i) monitor changes in Se_tot_ during ripening; (ii) optimize extraction and clean-up procedures for recovery of soluble Se compounds from cheese matrix; (iii) quantify free SeMet as a marker associated with ripening-related proteolytic changes; and (iv) determine the bioaccessible fraction of Se using an in vitro digestion model under different coagulant systems.

## 2. Materials and Methods

### 2.1. Cheesemaking

A total of 20 L of fortified raw milk (73 µg L^−1^ Se) obtained from cows fed high-selenium silage, as described previously [23], was transferred into a 50 L cheese vat and subjected to mild pasteurization at 73 °C for 30 s, followed by cooling to 30 °C. The milk was then inoculated with 2% (*w*/*w*) of a mesophilic aromatic starter culture (CHN-11; Chr. Hansen, Denmark; lyophilized form) containing *Lactococcus lactis* subsp. *cremoris*, *Lactococcus lactis* subsp. *lactis* biovar *diacetylactis*, *Lactococcus lactis* subsp. *lactis*, and *Leuconostoc* spp. The inoculated milk was divided into two equal portions (10 L each), each of which yielded a single cheese wheel, so that one wheel was produced per rennet type from the same pooled milk within one production run. The first portion was processed into Edam-type cheese using animal (calf) rennet (AR_Ch; Laktochym; 6 mL per 10 L of milk) and supplemented with CaCl_2_ (4 mL per 10 L). Coagulation was carried out at 30 °C for 60 min. The coagulum was then evaluated and cut using a cheese harp for 3 min to produce curd grains of approximately 3–4 mm. The curd was gently stirred for 5 min to promote whey separation, after which 3 L of whey was removed. The curd was again mixed at low speed. Curd washing was performed by adding 2 L of water preheated to 60 °C. The mixture was then gradually heated to a scalding temperature of 40 °C, maintained for 20 min under continuous stirring. All whey was subsequently drained, and dry salting was applied by adding 12 g of NaCl (equivalent to 1.0–1.5 kg per 1000 L of milk). The salted curd was mixed thoroughly and transferred into a pressing mold suitable for semi-hard cheeses. Pressing was performed using a pneumatic press (400 kPa; equivalent to a 120 kg force per pressure point) for 50 min. The pressed cheeses were left at ambient temperature for 12 h, cut into eight wedges, and coated with two layers of cheese wax (90–100 °C). The second portion of milk was processed analogously, using a microbial coagulant (MR_Ch; Fromase 220 TL BF; *Rhizomucor miehei* proteinase) at a dose of 3 mL per 10 L of milk. The resulting cheese yields, determined as fresh mass after pressing, were 1234 g per 10 L of milk (12.34%, *w*/*v*) for AR_Ch and 1065 g per 10 L of milk (10.65%, *w*/*v*) for MR_Ch. All cheeses were ripened under controlled conditions at 13 °C for 6 weeks.

### 2.2. Cheese Sampling and Characterization

Cheese samples produced with animal and microbial rennet were collected before ripening (week 0, W0) and at weekly intervals during the six-week ripening period, with one pre-allocated wedge withdrawn from each wheel at every time point and analyzed destructively. After carefully removing the wax coating, each cheese was portioned; one half was used for physicochemical analyses.

pH was measured using a pH meter (pHenomenal^®^ pH 1100 L, VWR, Radnor, PA, USA) equipped with a probe designed for cheese analysis. The electrode was inserted at three different points, and the mean value was recorded.

Fat content was determined using a butyrometer method. Approximately 3 g of grated and homogenized cheese was placed into a butyrometer and mixed with Van Gulik sulfuric acid (62%, *w*/*w*). After gentle shaking and heating in a 70 °C water bath until complete dissolution, 1 mL of amyl alcohol was added. The butyrometer was topped up with H_2_SO_4_, resealed, and gently mixed before centrifugation (1000 rpm, 5 min). Samples were then equilibrated at 65 °C for 5 min, and fat content was read directly from the butyrometer scale.

Dry matter was determined using a moisture analyzer (Kern DBS 60-3, Kern & Sohn, Balingen, Germany). Homogenized samples (≈0.8 g) were dried at 120 °C to constant weight. Fat in dry matter (FDM, %) was calculated as: *FDM* = (*t*/*S*) × 100, where *t* is fat content (%) and *S* is total dry matter (%).

### 2.3. Determination of Total Selenium

An aliquot of 120 mg of finely grated cheese was accurately weighed (±0.1 mg) and digested with 4.5 mL HNO_3_ (Analpure^®^, Analytika, Prague, Czech Republic) and 1.5 mL H_2_O_2_ (Rotipuran^®^, Carl Roth, Karlsruhe, Germany) using a closed-vessel microwave system (Discover SP-D, CEM, Matthews, NC, USA) at 180 °C for 15 min. The digests were diluted with Milli-Q water (≥18.2 MΩ·cm; Milli-Q system, Millipore, SAS, Molsheim, France) to a final volume of 100 mL. Selenium concentrations were determined by ICP-MS/MS (Agilent 8900, Agilent Technologies, Santa Clara, CA, USA) operated in mass-shift mode with oxygen, with the second quadrupole set to m/z 96 for monitoring ^80^Se^16^O^+^. Calibration was performed over the range 0.01–10 µg L^−1^ Se. Analyte signals were corrected using ^72^Ge^+^ and ^72^Ge^16^O^+^ as internal standards. For quality assurance, certified reference materials Bovine Liver (BCR^®^-185R) and Peach Leaves (SRM 1547, NIST), along with procedural blanks, were analyzed in parallel. The mean recovery of Se was 95 ± 5% (n = 5, BCR^®^-185R) and 109 ± 8% (n = 5, SRM 1547), demonstrating satisfactory accuracy and reproducibility of the analytical procedure. The procedural limit of detection (LOD) for Se_tot_ was 0.009 µg g^−1^ Se in cheese DM.

### 2.4. Evaluation of Extraction Method Robustness

The extraction of Se from cheese using ammonium acetate buffer was evaluated to assess method performance and robustness. Selected variables in the extraction scheme were modified, and their effects were monitored at defined parameter-change nodes (Table 1, including equipment specifications).

An aliquot of commercial Edam cheese, used as a surrogate matrix, was disintegrated (node_1: disintegration tool), homogenized, and weighed (±0.001 g) into 50 mL polypropylene (PP) tubes (node_2: cheese-to-extractant ratio, *m*/*v*). Subsequently, 30 mL of 20 mmol L^−1^ CH_3_COONH_4_ (p.a., Carl Roth, Karlsruhe, Germany) was added (node_3: initial buffer pH). The tubes were shaken on a reciprocating shaker (SHR-2D, Witeg, Wertheim, Germany) at 130 rpm for 15 min, briefly vortexed (10 s), and sonicated in an ultrasonic bath (node_4: sonication time). Samples were then either further shaken at 130 rpm (node_5: shaking time) or processed directly. Following centrifugation (C28-A, Boeco, Hamburg, Germany) at 3900× *g* for 10 min, 15 mL of the supernatant was transferred to a clean 50 mL PP tube.

Casein precipitation was induced by adjusting the pH to 4.60 ± 0.05 with 3.5 mol L^−1^ acetic acid (Analpure^®^, Analytika, Prague, Czech Republic). Samples were vortexed (10 s) and rapidly cooled in a −20 °C freezer to facilitate casein precipitation. The precipitate was separated by centrifugation at 3900× *g* for 10 min, and a 5 mL aliquot of the supernatant was transferred to a clean 15 mL PP tube. Hexane (Chromasolv™, Honeywell, Seelze, Germany) was then added (node_6: solvent addition). The mixture was shaken on a rotator (Multi Bio RS-24, Biosan, Riga, Latvia) at 30 rpm for 10 min and centrifuged (Rotina 320, Hettich, Tuttlingen, Germany) at 10,000× *g* for 10 min. From the aqueous phase, a 3 mL aliquot was acidified with 200 µL HNO_3_, passed through a 0.22 µm PES syringe filter, and analyzed for Se concentration by ICP-MS/MS.

### 2.5. Determination of Free Selenomethionine

Soluble selenium compounds were extracted from cheese samples in duplicate following the optimized acetate-buffer protocol established during method development. The final procedure, comprising nodes 1–6, is summarized in Figure S1. Ion-pairing reversed-phase high-performance liquid chromatography (IP-RP-HPLC; Agilent 1260, Agilent Technologies, Santa Clara, CA, USA) coupled to ICP-MS/MS was used to quantify SeMet, analyzed in duplicate, along with other Se species (selenocystine, Se-methylselenocysteine, selenite, and selenate; all purchased from Sigma-Aldrich, Steinheim, Germany) in cheese extracts. Chromatographic conditions and instrumental parameters for isocratic elution were adopted from [24]. The mobile phase consisted of 20 mmol L^−1^ ammonium acetate, 12 mmol L^−1^ (NH_4_)_2_SO_4_ (p.a., Lach:ner, Neratovice, Czech Republic), 1 mmol L^−1^ tetrabutylammonium hydroxide (40% aqueous solution, Carl Roth, Karlsruhe, Germany), and 2.5% (*v*/*v*) methanol (Rotisolv^®^, Carl Roth, Karlsruhe, Germany), adjusted to pH 5.2. The stability of SeMet during the entire analytical procedure was verified by spiking cheese samples with an aqueous SeMet solution, yielding a mean recovery of 88 ± 9%.

### 2.6. Selenium Bioaccessibility Assay

The bioaccessible fraction of Se (BA-Se) in cheese was estimated following the protocol of [25], which is based on the original method of [26]. In our adaptation, 5.00 ± 0.05 g of grated, homogenized cheese was used. The simulated salivary fluid (SSF) did not contain α-amylase. After extraction of the cheese bolus with SSF (10 mL), the liquid fraction was recovered but not analyzed. The same bolus was then successively extracted with simulated gastric fluid (SGF), prepared with porcine pepsin (10,000 FCC units mg^−1^, VWR, Radnor, PA, USA), and with simulated intestinal fluid (SIF), prepared with porcine pancreatin (8 × USP, Sigma-Aldrich, St. Louis, MO, USA) and bile (cholic acid, ≥45%, Carl Roth, Karlsruhe, Germany). The liquid fractions obtained from all three steps were pooled and analyzed for Se concentration by ICP-MS/MS. Prior to analysis, the final mixture was cooled (4 °C), centrifuged at 3900× *g* for 10 min, and a 1:3 aliquot was diluted with 2% HCl (Analpure^®^, Analytika, Prague, Czech Republic). The diluted samples were centrifuged again (3900× *g*, 10 min), filtered (0.22 µm, PES), and analyzed. All other parameters (salt concentrations, shaking, incubation times, and water bath temperature) followed [25].

### 2.7. Data Processing and Statistics

Results are reported as mean ± standard deviation (SD) from three replicates unless otherwise indicated. As a single cheesemaking batch was carried out per rennet type, the three replicate values obtained at each time point correspond to analytical (technical) replicates measured on the same wedge and do not represent independent cheese productions. For FDM, the uncertainty of the ratio of fat to dry matter was considered, while covariance between numerator and denominator was neglected. The study therefore follows a within-batch longitudinal design, and comparisons between the two rennet types are descriptive; the results should be regarded as exploratory. The regression models specified below were selected as empirical descriptions of the observed time courses, consistent with the gradual biochemical changes that accompany cheese ripening [16].

Selenium contents were modeled using weighted nonlinear least squares (weights = 1/SD^2^) with a one-phase exponential decay function with asymptote:

Y = *α* exp(−*kt*) + *γ* (1)

where *t* denotes ripening time, *α* is the initial offset above the asymptote, *k* is the rate constant, and *γ* is the long-term asymptotic level. Parameter ranges were restricted for stability (*α* ≥ 0, 0 < *k* ≤ 4, and *γ* was constrained within the observed data range).

Non-protein-bound SeMet contents were analyzed using weighted ordinary least squares regression with the general linear form:

Y = *β*_0_ + *β*_1_*t* (2)

where *β*_0_ denotes the intercept representing the initial level at week 0 and *β*_1_ is the slope corresponding to the net weekly increase in free SeMet content. Mean values per week were fitted with inverse variance weighting (1/SD^2^) to account for heteroscedastic replicate uncertainty.

Bioaccessible Se fractions (expressed as % of total Se) were fitted with an asymptotic exponential growth model:

Y = *α* + (*γ* − *α*)[1 − exp(−*kt*)] (3)

using the same parameter notation as above with additional restrictions (*α* < 65, 0 < *k* ≤ 4). This relative expression allowed direct comparability between cheeses, with the understanding that total Se content itself decreased slightly during ripening.

For the robustness assessment of the extraction procedure (Section 2.4), the three replicates at each condition (node) were independent extractions performed on a commercial cheese used as a surrogate matrix and were therefore treated as independent samples. Given the unequal within-group variances observed across conditions, differences in Se extraction efficiency were evaluated using one-way Welch’s ANOVA followed by the Games–Howell post-hoc test for pairwise comparisons, both of which are robust to heteroscedasticity. Statistical significance was set at *p* < 0.05.

The presented data were analyzed using SPSS Statistics (v29.0; IBM, Armonk, NY, USA) and R (v4.2.2; R Foundation for Statistical Computing, Vienna, Austria).

## 3. Results

### 3.1. Physicochemical Characteristics of Cheeses During Ripening

**Figure 1:**
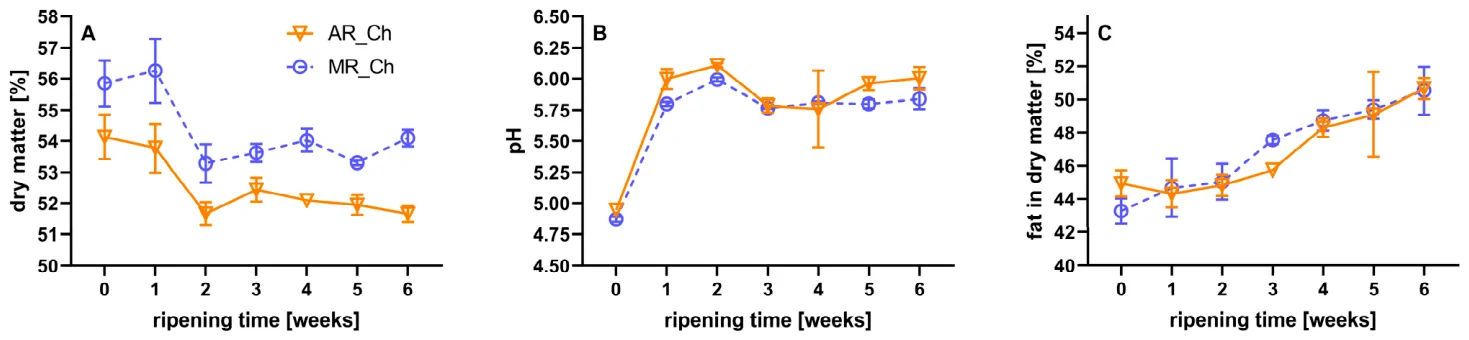
Changes in selected physicochemical parameters during ripening of cheeses coagulated with animal rennet (AR_Ch) and microbial rennet (MR_Ch): (**A**) dry matter; (**B**) pH; (**C**) fat in dry matter (FDM). Error bars represent the mean ± SD of three analytical replicates measured on the same cheese wedge.

Dry matter in AR_Ch was 54.0 ± 0.7% during the initial ripening period (W0–W1; Figure 1A), then dropped at W2 and remained stable at 52.0 ± 0.4% for the rest of the trial. In MR_Ch, DM content was consistently higher by about 2 percentage points at all sampling times, reaching 54.1 ± 0.3% at W6.

The pH profiles of both cheeses followed a nearly identical pattern during ripening (Figure 1B). Values started at 4.91 ± 0.04 at W0, peaked at 6.0–6.1 at W2, and declined slightly thereafter. By W6, MR_Ch tended to be marginally more acidic (pH 5.84 ± 0.08) compared with AR_Ch (pH 6.00 ± 0.09).

Figure 1C shows changes in FDM during ripening. In both cheeses, fat content exhibited only limited variation during the early ripening phase. From W3 onwards, fat levels increased steadily, with no apparent differences between AR_Ch and MR_Ch in most samples. The mean FDM of the final cheeses was 50–51%.

### 3.2. Total Selenium Content During Ripening

**Figure 2:**
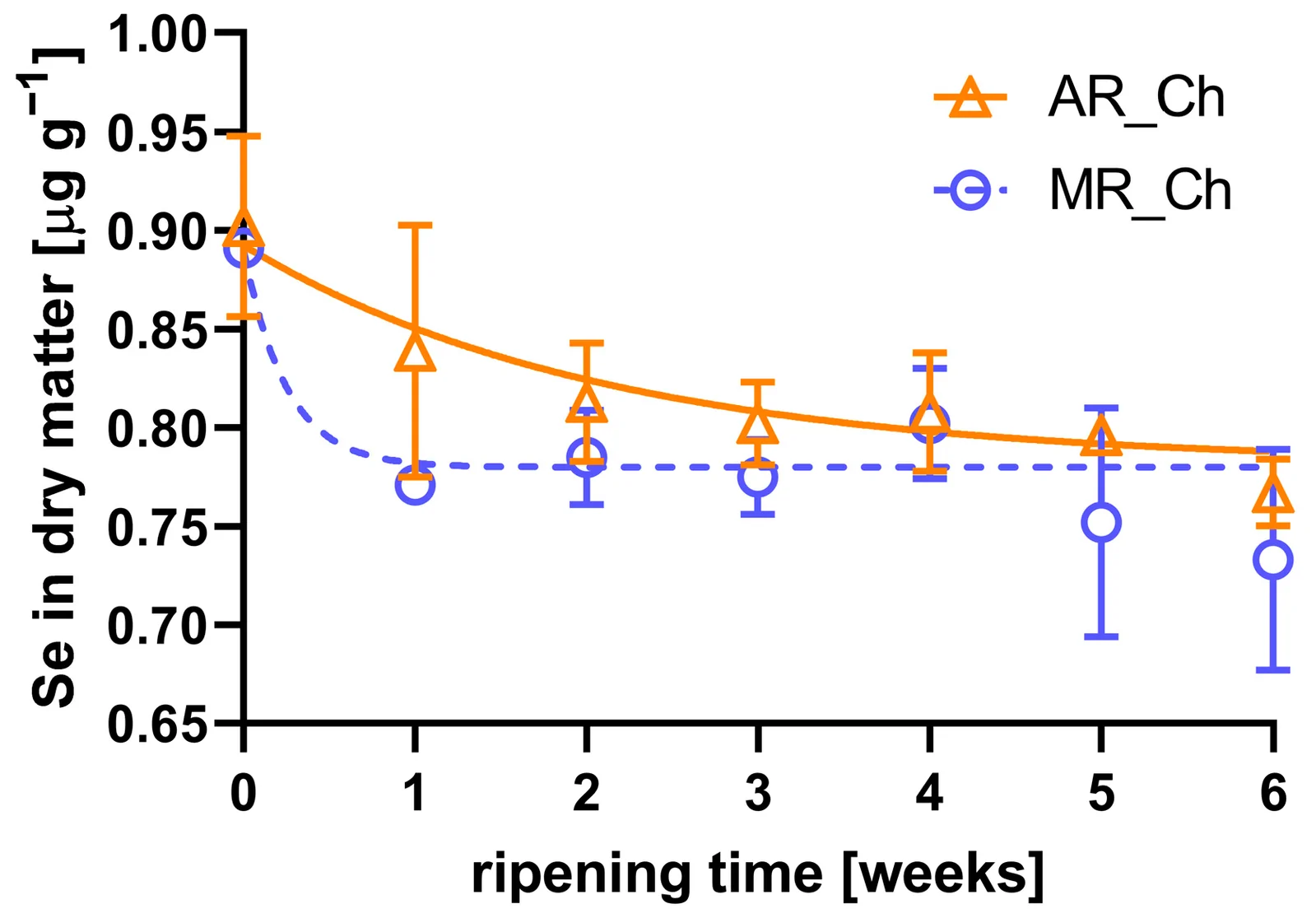
Changes in total selenium content during six weeks of ripening in cheeses coagulated with animal rennet (AR_Ch) and microbial rennet (MR_Ch). Curves represent fitted one-phase exponential decay models. Error bars represent the mean ± SD of three analytical replicates measured on the same cheese wedge.

The total Se contents in fresh curds of both cheeses did not differ, measuring 0.90 ± 0.05 µg g^−1^ (AR_Ch) and 0.89 ± 0.01 µg g^−1^ (MR_Ch) on a DM basis (Figure 2). At W1, a marked decrease of 13.5% was observed in MR_Ch. From W2 onwards, Se content stabilized at 0.78 ± 0.01 µg g^−1^, but declined slightly toward the end of the trial, reaching 0.73 ± 0.06 µg g^−1^ by W6. In AR_Ch, only a modest decrease (7.0%) was observed at W1.

Thereafter, Se content showed a gradual decline throughout ripening, reaching 0.77 ± 0.02 µg g^−1^ by W6. Nonlinear regression (parameter estimates in Table S1) confirmed that both cheeses converge toward similar long-term Se levels (*γ* ≈ 0.77–0.78 µg g^−1^). MR_Ch showed a sharp early drop, whereas Ar_Ch exhibited a gradual decline. The fitting was adequate, with a weighted R^2^ of 0.75 (AR_Ch) and 0.99 (MR_Ch), indicating that the model captured the observed patterns well.

### 3.3. Robustness and Efficiency of Selenium Extraction

**Figure 3:**
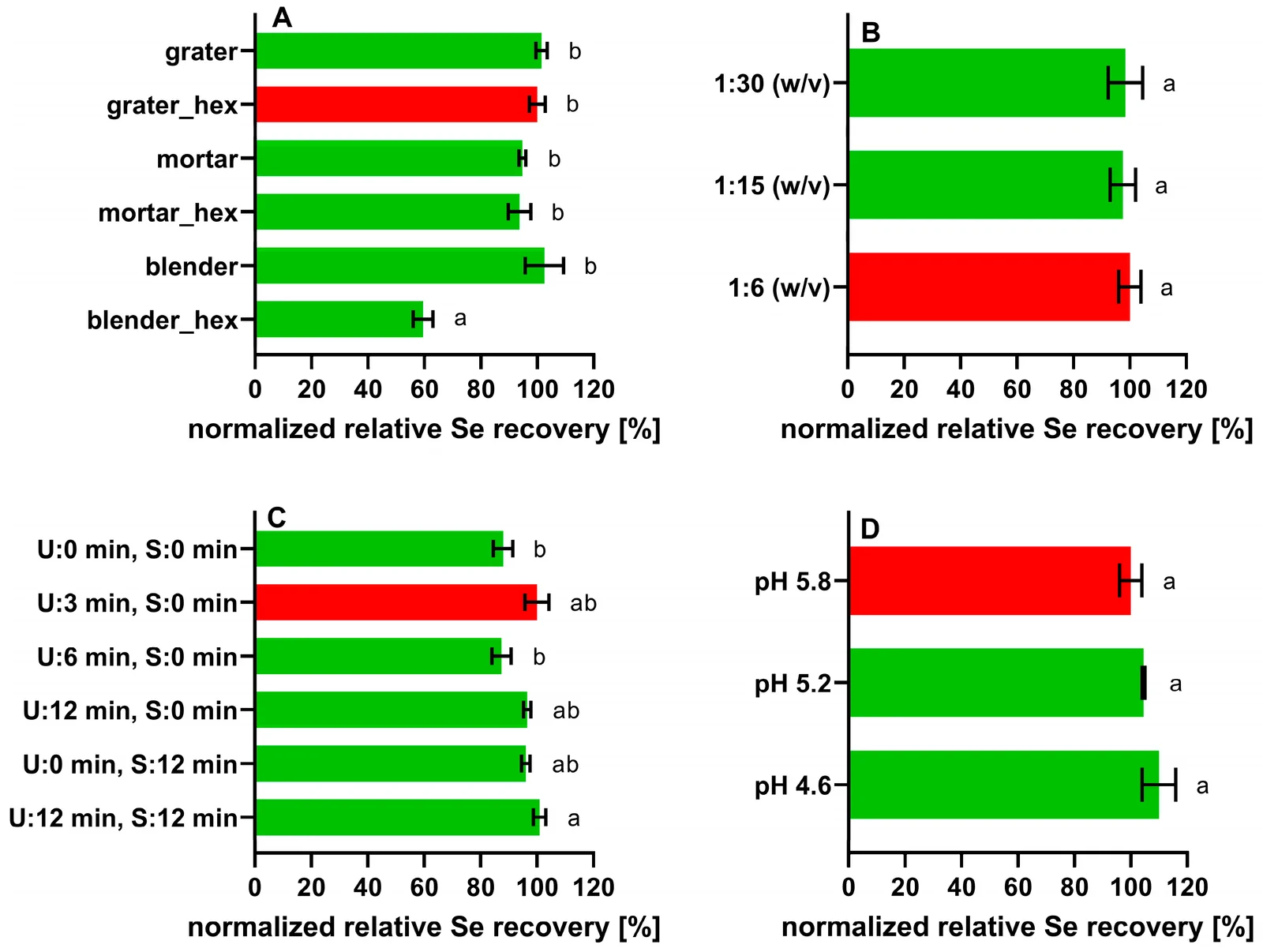
Effects of extraction and clean-up parameters on normalized selenium recovery from a commercial Edam cheese used as a surrogate matrix. Normalization refers to the recovery obtained for the reference variant within each parameter set (highlighted in red), not to the total Se content of the cheese: (**A**) disintegration tool (grater, mortar, blender); (**B**) solid-to-liquid ratio; (**C**) extraction conditions (U = sonication time; S = additional shaking time); (**D**) initial buffer pH. Bars represent means ± SD of three independent extractions. Different letters next to the bars indicate statistically significant differences (Welch’s one-way ANOVA followed by Games–Howell post-hoc test, *p* < 0.05).

Robustness of the extraction and clean-up steps was assessed to prevent small procedural variations from affecting Se recovery. Tool selection for cheese disintegration (grater, mortar, blender) had only a minor effect on Se release (Figure 3A). However, when followed by hexane clean-up, mortar (93.8 ± 4.0%) and blender (59.5 ± 3.5%) showed reduced recoveries and presented cleaning difficulties under wet conditions; therefore, grating with hexane clean-up was adopted for the final protocol. The solid-to-liquid ratio did not influence Se release (Figure 3B), and the narrowest tested ratio (1:6 *w*/*v*) was selected to optimize detection, yielding a procedural LOD of 0.0002 µg g^−1^ (fresh matter) for the Se speciation analysis method. Sonication time beyond 3 min, or additional post-sonication shaking, did not enhance recovery (Figure 3C); hence, the protocol employed 3 min sonication only. Finally, variation in the initial pH of 20 mmol L^−1^ ammonium acetate had no pronounced effect on recovery (Figure 3D). The final method applied pH 5.8 to remain above the casein isoelectric point, ensuring that protein precipitation occurred later during clean-up.

**Figure 4:**
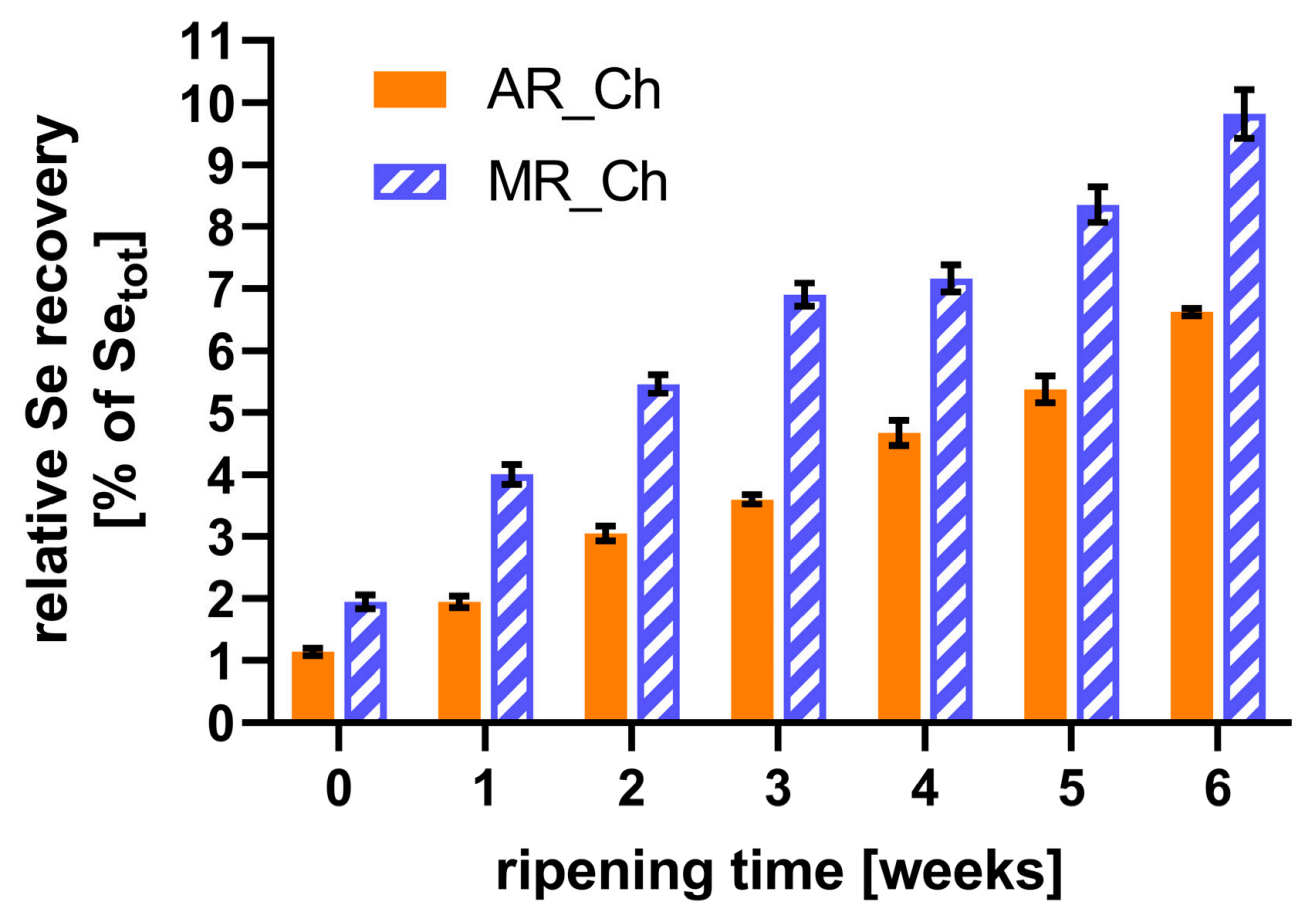
Efficiency of selenium extraction under optimized conditions, expressed relative to total selenium (Se_tot_) during six weeks of ripening in cheeses coagulated with animal rennet (AR_Ch) and microbial rennet (MR_Ch). Error bars represent the mean ± SD of three analytical replicates measured on the same cheese wedge.

Figure 4 illustrates the efficiency of Se extraction under optimized conditions, expressed relative to Se_tot_ during ripening. These data also serve as a critical reference for Se speciation analysis, as they define the input Se levels for subsequent HPLC-ICP-MS/MS measurements. In both cheeses, Se recovery rose steadily without reaching a plateau, indicating increasing Se availability for subsequent speciation analysis. In fresh curd, the proportion of Se recovered was 1.14 ± 0.06% (AR_Ch) and 1.95 ± 0.11% (MR_Ch). These recoveries increased five- to sixfold by W6, while distinct differences between the two cheeses were maintained throughout the ripening period.

### 3.4. Free Selenomethionine Dynamics During Ripening

**Figure 5:**
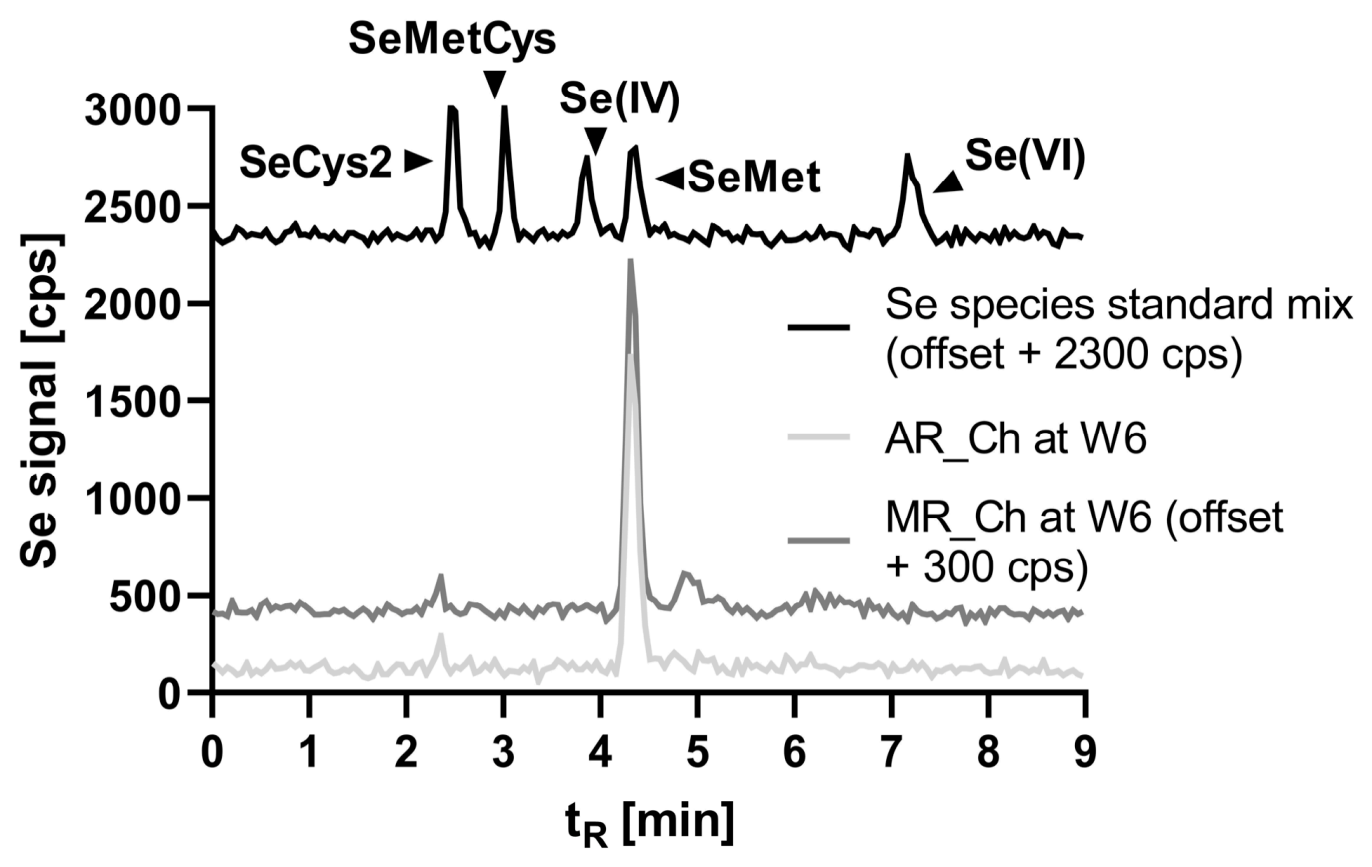
Chromatograms of selenium species recovered in acetate extracts. The upper chromatogram shows the standard mixture containing 0.1 µg L^−1^ Se of each individual species: selenocystine (SeCys2), Se-methylselenocysteine (SeMetCys), selenite (SeIV), selenomethionine (SeMet), and selenate (SeVI). The lower chromatograms show the aqueous extracts from cheeses after 6 weeks of ripening, coagulated with animal rennet (AR_Ch) or microbial rennet (MR_Ch). Chromatograms are vertically offset as indicated for clarity.

Chromatograms of Se species recovered in acetate extracts during ripening are presented in Figure 5 and Figure S2.

**Figure 6:**
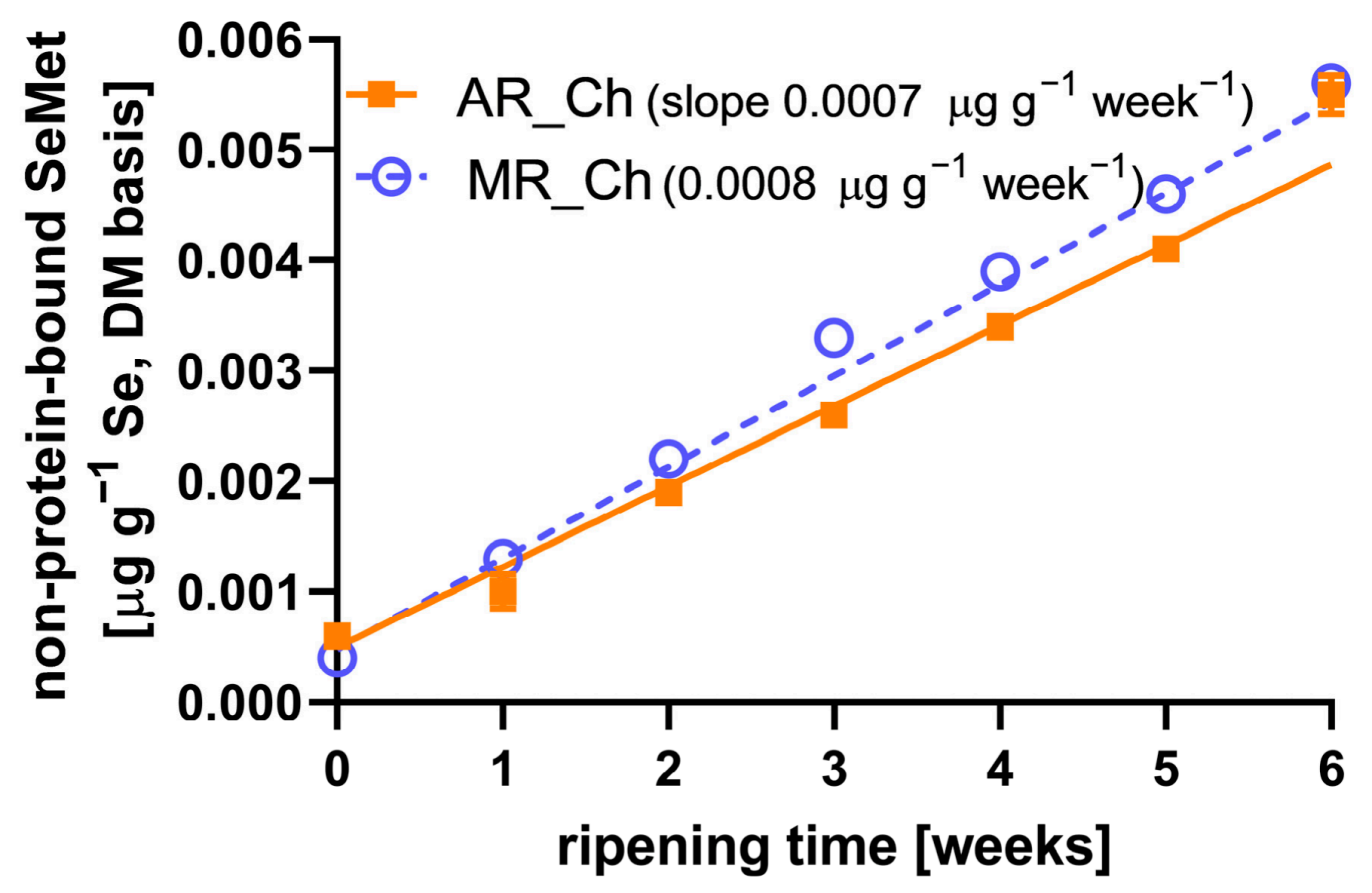
Changes in free selenomethionine (SeMet) content during 6 weeks of ripening in cheeses coagulated with animal rennet (AR_Ch) and microbial rennet (MR_Ch). Lines represent fitted linear regression models. Error bars represent the mean ± SD of two analytical replicates measured on the same cheese wedge.

Among the Se species, only SeMet could be reliably identified, as confirmed by retention time matching and spiking with an aqueous standard. The mean recovery of SeMet spiked at 0.003 µg Se g^−1^ cheese (fresh matter basis) was 86 ± 5%. In fresh curds, SeMet levels (Figure 6) were as low as 0.0004 ± 0.0001 µg g^−1^ Se (MR_Ch) and 0.0006 ± 0.0001 µg g^−1^ Se (AR_Ch), values close to the method LOD on a DM basis, corresponding to only 0.05–0.06% of Se_tot_. Both cheeses showed a continuous linear increase in free SeMet during ripening, with no indication of plateauing (R^2^ ≥ 0.991; Table S2). By W6, free SeMet reached ~0.006 µg g^−1^ Se in both cheeses, accounting for 0.72–0.77% of Se_tot_. The regression slopes were 0.0007 µg g^−1^ week^−1^ for AR_Ch and 0.0008 µg g^−1^ week^−1^ for MR_Ch, suggesting nearly identical rates of free SeMet increase.

### 3.5. In Vitro Bioaccessibility of Selenium During Ripening

**Figure 7:**
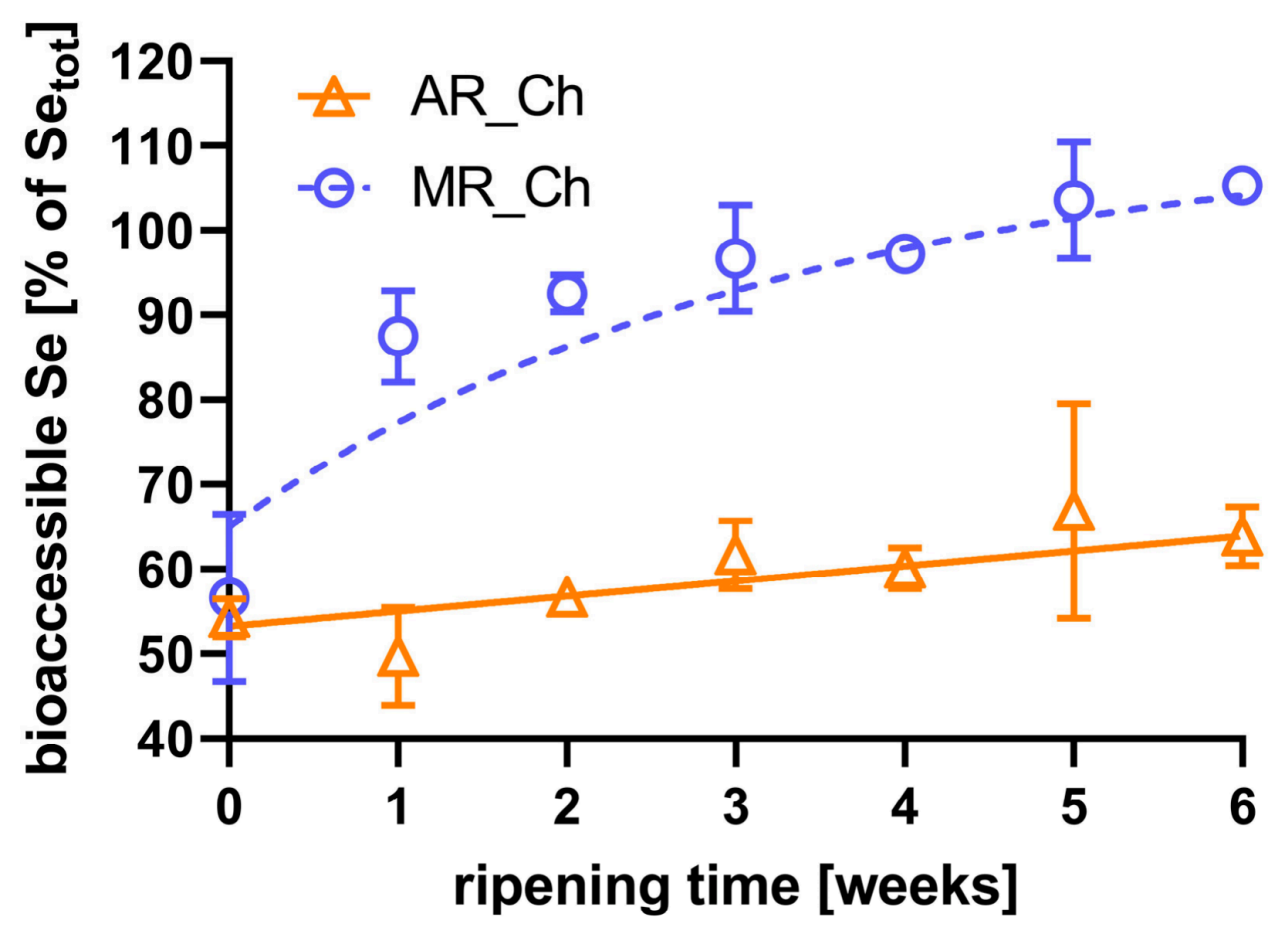
Estimation of bioaccessible selenium by an in vitro gastrointestinal digestion model, expressed relative to total selenium (Se_tot_), in cheeses coagulated with animal rennet (AR_Ch) and microbial rennet (MR_Ch). Curves represent fitted asymptotic exponential growth models. Error bars represent the mean ± SD of three analytical replicates measured on the same cheese wedge.

Bioaccessible Se (Figure 7) accounted for a considerable fraction already in fresh curds, 54.2 ± 2.3% of total Se in AR_Ch and 56.6 ± 9.9% in MR_Ch. In MR_Ch, the fraction increased markedly to 87.5 ± 5.4% at W1 and then stabilized at 101 ± 4% during W3–W6.

In contrast, AR_Ch showed no abrupt rise, with values remaining relatively stable throughout ripening (59.0 ± 5.9% overall; 63.9 ± 3.5% at W6). The MR_Ch data were adequately captured by an exponential saturation model (R^2^ = 0.81; Table S3). The asymptotic estimate slightly exceeded 100% (112%), likely reflecting fitting-related uncertainty near the upper boundary of an operationally defined assay. Experimentally observed values nevertheless stabilized close to quantitative Se recovery from week 3 onwards. In contrast, although AR_Ch yielded a comparable R^2^ (0.83), the fitted asymptote could not be interpreted reliably because of boundary effects, and the dataset was therefore evaluated descriptively.

## 4. Discussion

The trends in physicochemical parameters (DM, FDM, and pH) observed during the ripening of the lab-scale cheeses (Figure 1A–C) reflected typical maturation events. Fat became slightly more concentrated in DM as proteolysis progressed, while pH increased due to lactic acid degradation, proteolysis, and ammonia release, reaching up to 0.5 units above values usually reported for Dutch-type semi-hard cheeses [12,20]. Both cheeses showed a minor decrease in DM after W1, likely resulting from salt diffusion and proteolysis, which promote water retention in the matrix. The initial discrepancy in DM between AR_Ch (54%) and MR_Ch (56%) curds at W0 may be explained by the distinct coagulation agents used.

After 6 weeks of ripening, both AR_Ch and MR_Ch qualified for the nutrition claim “*high in selenium*” [27], providing 40 µg Se per 100 g fresh matter, equivalent to 73% of the recommended daily allowance [28]. Despite this, both cheeses showed a decline in Se_tot_, with losses of 15% in AR_Ch and 18% in MR_Ch by W6 (Figure 2). Modeling suggested that the main difference between the cheeses lay in their early dynamics, with MR_Ch showing a sharp initial decrease by W1 followed by stabilization. To our knowledge, Se loss during cheese ripening has not been documented previously. Reports on Se dynamics in cheese are limited and inconsistent. Ianni et al. [21] found no change in Se content of Se-enriched Caciocavallo between 7 and 120 days, while a market survey of commercial Parmigiano Reggiano reported higher Se_tot_ in cheeses ripened for 40 months compared with 24 months [29].

Selenium depletion is plausible due to its ability to form volatile species. Given the chemical similarity between Se and S, since both sit in Group 16 of the periodic table, and the fact that starter lactic acid bacteria (SLAB) are well-known producers of volatile S compounds from methionine (Met) and cysteine (Cys) catabolism [30], there may exist comparable pathways for Se transformation. Thompson-Eagle and Frankenberger [31] also showed that peptides from hydrolyzed casein stimulated Se biomethylation in natural waters, suggesting that proteolysis could provide substrates for SLAB to metabolize Se. Although microbial volatilization is a likely mechanism, it cannot fully explain the sharp 13% decline in Se_tot_ observed in MR_Ch between W0 and W1. This rapid loss is more likely linked to cheesemaking technology. The greater early decline in MR_Ch indicates that microbial rennet enzymes were more active during initial proteolysis. This interpretation is consistent with reports that microbial coagulants often display high proteolytic activity but low specificity [19,32,33]. Soon after coagulant addition, such activity can promote the formation of casein breakdown fragments that are lost with whey during the initial stages of ripening. It also agrees with [18], who compared Edam cheeses ripened for 90 days and found that microbial rennet (*R. miehei*) induced higher water-soluble nitrogen and more extensive degradation of αs- and β-caseins than calf rennet. Our interpretation is further supported by the lower fresh cheese yield of MR_Ch, which was 13.7% below that of AR_Ch, and by the higher efficiency of Se release from the MR_Ch matrix under optimized aqueous extraction (Figure 4).

Total free amino acids (FAAs) are widely used as indicators of cheese ripening [20,34,35]. However, individual FAAs often display non-linear patterns, limiting their reliability as universal ripening indices [36]. In the present study, free SeMet exhibited a consistent linear increase across both cheese variants during the 6-week ripening period (Figure 6), suggesting that it may serve as a useful indicator of ripening-associated proteolytic changes within the extractable Se fraction. The observed trend also highlights the sensitivity of the applied analytical approach to subtle biochemical changes occurring during cheese maturation. To our knowledge, this is the first report on SeMet dynamics in cheese; its accumulation and catabolism remain largely unexplored. By analogy, Met has been reported to rise with ripening [22,35], although it is also among the most variable FAAs, reflecting its susceptibility to metabolic transformations [30]. The similar rates of SeMet increase in both cheeses, irrespective of the coagulant used (Figure 6), indicate comparable peptidase activity and support the view that rennet activity is largely confined to the primary proteolysis of casein into larger peptides [16,17]. This further implies that secondary proteolysis proceeded under substrate-saturated conditions. However, benchmark studies have shown that FAA accumulation can be influenced not only by residual rennet activity but also by rennet type [32,34]. For instance, El-Aidie et al. [18] reported that free Met in Edam cheese was 61% higher when coagulated with *R. miehei* compared with calf rennet after 90 days of ripening. In contrast, and in agreement with our observations, D’Incecco et al. [33] reported no effect of coagulant type on FAA accumulation in hard cheeses after 9–18 months of ripening. Finally, the absence of a plateau or decline in SeMet content suggests that its catabolism was kinetically constrained, implying that any degradation proceeded at a slower rate than its release during ripening. This observation may also be of biochemical interest, since sulfur-containing amino acids such as Met are known precursors of volatile compounds involved in cheese flavor development through microbial catabolic pathways [30]. Although SeMet likely contributes only marginally relative to native sulfur amino acids in cheese, its structural similarity to Met suggests that Se-containing metabolites may participate in aroma-forming pathways during cheese maturation.

The selenium content of a food product provides limited nutritional insight unless complemented by data on bioaccessibility, i.e., the fraction of Se released from the matrix and potentially available for intestinal absorption [26]. In vitro gastrointestinal digestion models have therefore gained popularity for assessing trace element bioaccessibility from complex food matrices. Reported BA-Se values vary substantially among foods and experimental protocols. For example, BA-Se fractions in maize range from 22 to 90%, depending on the sample and digestion model applied [37]. Although operationally defined, such models are particularly valuable when applied within a single study to evaluate technological or compositional factors influencing Se release, including Se fortification level [25] and culinary processing [37]. To our knowledge, no studies have examined BA-Se in cheese during ripening. However, the suitability of in vitro gastrointestinal models for dairy matrices was demonstrated by [38], who reported efficient cleavage of Se-containing milk proteins into amino acids and peptides <1.5 kDa in Se-enriched fermented milk.

Our results demonstrated distinct BA-Se dynamics between cheeses manufactured with different coagulants (Figure 7). In MR_Ch, Se remained almost completely bioaccessible throughout ripening, suggesting that microbial rennet facilitated extensive Se release during simulated gastrointestinal digestion. This behavior likely reflects the proteolytic profile of the *R. miehei* aspartic protease, which differs from calf chymosin in two complementary respects. First, it hydrolyzes κ-casein and other caseins at multiple sites, rather than acting almost exclusively at the Phe_105_–Met_106_ bond [39] and references therein. Second, it displays substantially higher heat stability; a larger fraction of its activity therefore survives coagulation and cooking, remains in the curd, and contributes to extensive proteolysis during ripening [16]. Microbial rennet thus generates a more hydrolyzed casein network with a higher proportion of small peptide fragments, which provides digestive enzymes with pre-cleaved substrates and enables near-quantitative release of Se during simulated gastrointestinal digestion. The importance of ripening for the fate of cheese components during gastrointestinal digestion has been demonstrated in Ras cheese, where the peptide profile released upon INFOGEST digestion, and the resulting biological activity after ingestion, varied with ripening time [40]. In contrast, AR_Ch showed a flatter pattern and lower overall BA-Se, stabilizing at 64–67% by W5–W6. These differences may be nutritionally relevant, since the extent of Se release from the cheese matrix likely influences the fraction potentially available for intestinal uptake. Notably, MR_Ch exhibited a sharp rise in BA-Se between W0 (57%) and W1 (88%), accompanied by a pronounced loss of Se_tot_ (Figure 2), both consistent with intensive primary proteolysis.

Overall, in the context of Se-enriched cheese production, applying coagulants with broad proteolytic activity (e.g., from *R. miehei*) may be a double-edged strategy, potentially lowering Se retention in the cheese matrix during ripening while improving Se bioaccessibility.

## 5. Conclusions

Edam-type cheeses manufactured at laboratory scale from Se-enriched milk (73 µg L^−1^ Se) provided roughly 40 µg Se per 100 g portion after six weeks of ripening, irrespective of rennet type. Such a level meets the EU criteria for the nutrition claim ‘high in selenium.’ Storage, however, was associated with Se depletion, and the extent of loss differed between the two coagulants studied. In particular, microbial rennet (*R. miehei*) was associated with an early 13.5% reduction in Se_tot_, possibly reflecting its more intense and less specific casein cleavage during primary proteolysis. This may enhance Se release from the matrix and promote leaching with soluble polypeptides. The aqueous extraction approach enabled sensitive monitoring of free SeMet dynamics within the soluble Se fraction during cheese ripening. Quantification of free SeMet as Se equivalents down to 0.0003 µg g^−1^ revealed a consistent linear increase during ripening in both cheeses. Within the studied system, free SeMet may therefore represent a useful indicator of ripening-associated proteolytic changes. Finally, in vitro digestion suggested that the choice of coagulant may influence Se bioaccessibility, with microbial rennet yielding considerably higher values than calf rennet. Confirmation across multiple independent cheesemaking batches would be needed to establish coagulant-driven effects with statistical support and to verify the generalizability of the present findings. Future research should also investigate the contribution of starter and nonstarter microbial cultures, which are likely to shape secondary proteolysis and SeMet catabolism.

## Figures and Tables

**Table 1:** Extraction variables and parameter-change nodes for Se recovery from cheese matrix.

Nodes	Factors	Codes	Factor Levels
1	disintegration tool	DT1; DT2; DT3	grater ^a^; mortar ^b^; blender ^c^
2	solid-to-liquid ratio	SL1; SL2; SL3	1:30; 1:15; 1:6 (*w*/*v*)
3	initial buffer pH	pH1; pH2; pH3	5.8; 5.2; 4.6
4	sonication time	U1; U2; U3; U4	0 min; 3 min; 6 min; 12 min ^d^
5	additional shaking	S1; S2	0 min; 12 min ^e^
6	hexane addition	HEX1; HEX2	0 mL; 2 mL

^a^ stainless-steel grater (aperture ≈ 1 mm); ^b^ porcelain mortar and pestle; ^c^ household kitchen blender (Basto, ETA, Hlinsko, Czech Republic; 400 W); ^d^ ultrasonic bath (40 kHz, 120 W); ^e^ reciprocating shaker SHR-2D (Witeg, Wertheim, Germany; 130 rpm).

## Data Availability

Dataset available on request from the authors.

## Supplementary Materials

The following supporting information can be downloaded at: https://www.mdpi.com/article/10.3390/foods15173123/s1, Figure S1: Workflow of the final aqueous extraction protocol for selenium from cheese matrix prior to HPLC–ICP–MS/MS analysis; Figure S2: Chromatograms of selenium species in aqueous cheese extracts obtained with 20 mmol L^−1^ ammonium acetate (pH 5.8) from cheeses coagulated with microbial rennet (MR_Ch) during ripening (weeks 0–6); Table S1: Parameter estimates of the weighted nonlinear regression (one-phase exponential decay with asymptote) describing selenium contents in cheeses coagulated with animal rennet (AR_Ch) and microbial rennet (*Rhizomucor miehei*; MR_Ch) during ripening (weeks 0–6); Table S2: Parameter estimates of the weighted ordinary least squares regression describing non-protein-bound SeMet contents in cheeses coagulated with animal rennet (AR_Ch) and microbial rennet (*Rhizomucor miehei*; MR_Ch) during ripening (weeks 0–6); Table S3: Parameter estimates of the weighted nonlinear least squares regression (asymptotic exponential growth model) describing bioaccessible selenium fractions (expressed as % of total Se) in cheeses coagulated with animal rennet (AR_Ch) and microbial rennet (*Rhizomucor miehei*; MR_Ch) during ripening (weeks 0–6).
