# Supplementary material for: Effect of Rennet Type on Selenium Retention and Its In-Vitro Bioaccessibility in Se-Enriched Edam Cheese: An Exploratory Study
Source: Foods. 2026 Sep 2;15(17):3123. doi: 10.3390/foods15173123 (PMC13565477; doi:10.3390/foods15173123)
Supplement: Supplementary file 1 [file foods-15-03123-s001.zip › foods-4374602-supplementary.pdf]

Supplementary Materials

Effect of rennet type on selenium retention and its in-vitro bioaccessibility in Se-enriched Edam cheese: an exploratory study

Lukáš Praus [1,*], Veronika Legarová [2], Lucie Kejdová Rysová [2], Michal Šrámek [1], Lukáš Kaplan [1], Ondřej Sedlář [1], Pavel Tlustoš [1] and Jiřina Száková [1]

[1] Department of Agroenvironmental Chemistry and Plant Nutrition, Czech University of Life Sciences, Prague, Kamýcká 129, 165 00, Praha-Suchdol, Czech Republic

[2] Department of Food Science, Czech University of Life Sciences, Prague, Kamýcká 129, 165 00, Praha-Suchdol, Czech Republic

*Corresponding author: prausl@af.czu.cz

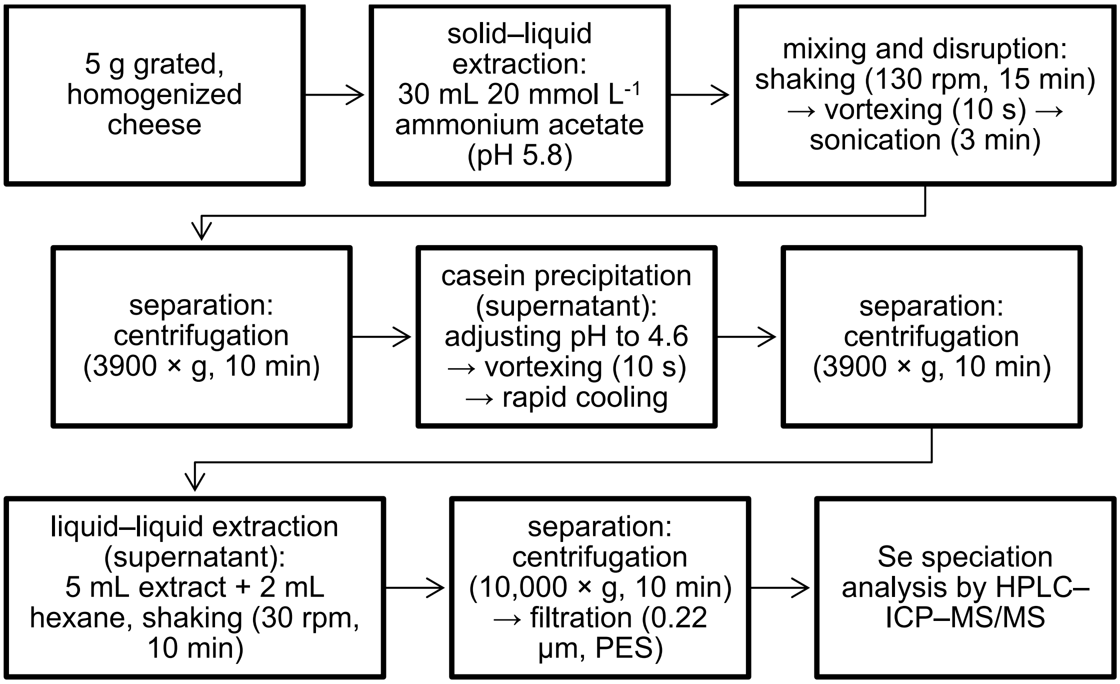

Figure S1. Workflow of the final aqueous extraction protocol for selenium from cheese matrix prior to HPLC–ICP–MS/MS analysis.

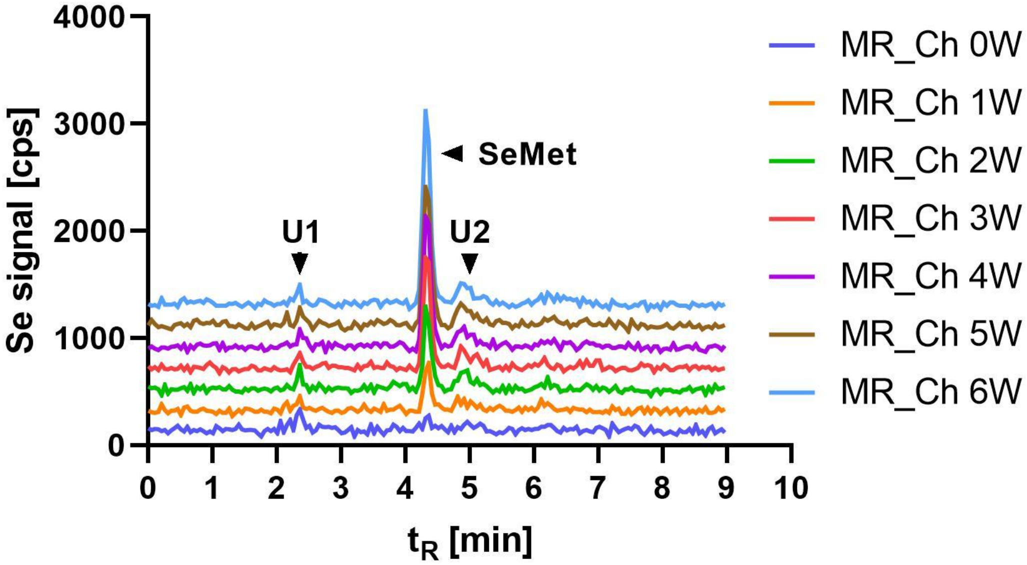

Figure S2. Chromatograms of selenium species in aqueous cheese extracts obtained with 20 mmol L^{-1} ammonium acetate (pH 5.8) from cheeses coagulated with microbial rennet (MR_Ch) during ripening (weeks 0–6). Selenomethionine (t_R = 4.3 min) was the predominant species, accompanied by unidentified Se compounds (U1 and U2) at 2.4 and 4.9 min. Chromatograms are vertically offset for clarity.

Table S1. Parameter estimates of the weighted nonlinear regression (one-phase exponential decay with asymptote) describing selenium contents in cheeses coagulated with animal rennet (AR_Ch) and microbial rennet (*Rhizomucor miehei*; MR_Ch) during ripening (weeks 0–6). The model was fitted as $Y = \alpha \exp(-kt) + \gamma$ with weights = $1/SD^2$. γ values represent best-fit estimates with 95% confidence intervals (CI).

Cheese	α	k	γ	γ (95% CI)	R^2 weighted	WSS
AR_Ch	0.111	0.476	0.782	0.713–0.850	0.752	2.286
MR_Ch	0.122	4.000	0.769	0.734–0.804	0.989	55.65

Note: t denotes ripening time; α is the initial offset above the asymptote; k is the rate constant; and γ is the long-term asymptotic level. Parameter ranges were restricted for stability ($\alpha \geq 0$; $0 < k \leq 4$; γ constrained within the observed data range).

Table S2. Parameter estimates of the weighted ordinary least squares regression describing non-protein-bound SeMet contents in cheeses coagulated with animal rennet (AR_Ch) and microbial rennet (*Rhizomucor miehei*; MR_Ch) during ripening (weeks 0–6). The model was fitted as $Y = \beta_0 + \beta_1 t$ with weights = $1/SD^2$.

Cheese	β_0	β_1	R^2 weighted

AR_Ch	0.00050	0.00073	0.991
MR_Ch	0.00048	0.00083	1.000

Note: t denotes ripening time; β_0 is the intercept representing the initial level at week 0; and β_1 is the slope corresponding to the net weekly increase in free SeMet content.

Table S3. Parameter estimates of the weighted nonlinear least squares regression (asymptotic exponential growth model) describing bioaccessible selenium fractions (expressed as % of total Se) in cheeses coagulated with animal rennet (AR_Ch) and microbial rennet (*Rhizomucor miehei*; MR_Ch) during ripening (weeks 0–6). The model was fitted as $Y = \alpha + (\gamma - \alpha)[1 - \exp(-kt)]$ with weights = $1/SD^2$.

Cheese	α	k	γ	R^2 weighted	WSS
AR_Ch	53.22	0.012	–	0.831	1.810
MR_Ch	65.00	0.306	111.53	0.806	16.060

Note: notation follows that in Table S1, with additional restrictions ($\alpha < 65$; $0 < k \leq 4$).
